# Chromosome-Level Genome Assembly of the American Cranberry (*Vaccinium macrocarpon* Ait.) and Its Wild Relative *Vaccinium microcarpum*

**DOI:** 10.3389/fpls.2021.633310

**Published:** 2021-02-10

**Authors:** Luis Diaz-Garcia, Luis Fernando Garcia-Ortega, Maria González-Rodríguez, Luis Delaye, Massimo Iorizzo, Juan Zalapa

**Affiliations:** ^1^Instituto Nacional de Investigaciones Forestales, Agrícolas y Pecuarias, Campo Experimental Pabellón, Aguascalientes, Mexico; ^2^Department of Genetic Engineering, Cinvestav Unidad Irapuato, Irapuato, Guanajuato, Mexico; ^3^Plants for Human Health Institute, North Carolina State University, Kannapolis, NC, United States; ^4^Department of Horticulture, University of Wisconsin, Madison, WI, United States; ^5^USDA-ARS, Vegetable Crops Research Unit, University of Wisconsin, Madison, WI, United States

**Keywords:** American cranberry, *Vaccinium*, genome duplication, genome evolution, anthocyanin biosynthesis

## Abstract

The American cranberry (*Vaccinium macrocarpon* Ait.) is an iconic North American fruit crop of great cultural and economic importance. Cranberry can be considered a fruit crop model due to its unique fruit nutrient composition, overlapping generations, recent domestication, both sexual and asexual reproduction modes, and the existence of cross-compatible wild species. Development of cranberry molecular resources started very recently; however, further genetic studies are now being limited by the lack of a high-quality genome assembly. Here, we report the first chromosome-scale genome assembly of cranberry, cultivar Stevens, and a draft genome of its close wild relative species *Vaccinium microcarpum*. More than 92% of the estimated cranberry genome size (492 Mb) was assembled into 12 chromosomes, which enabled gene model prediction and chromosome-level comparative genomics. Our analysis revealed two polyploidization events, the ancient γ-triplication, and a more recent whole genome duplication shared with other members of the Ericaeae, Theaceae and Actinidiaceae families approximately 61 Mya. Furthermore, comparative genomics within the *Vaccinium* genus suggested cranberry-*V. microcarpum* divergence occurred 4.5 Mya, following their divergence from blueberry 10.4 Mya, which agrees with morphological differences between these species and previously identified duplication events. Finally, we identified a cluster of subgroup-6 R2R3 MYB transcription factors within a genomic region spanning a large QTL for anthocyanin variation in cranberry fruit. Phylogenetic analysis suggested these genes likely act as anthocyanin biosynthesis regulators in cranberry. Undoubtedly, these new cranberry genomic resources will facilitate the dissection of the genetic mechanisms governing agronomic traits and further breeding efforts at the molecular level.

## Introduction

The American cranberry (*Vaccinium macrocarpon* Ait.) is a diploid (2n = 2x = 24), woody perennial fruit crop well adapted to the acidic bogs of North America (Eck, 1990). As one of only three native fruit species commercially grown in the United States, cranberry generates more than $3.5 billion in economic value, which in a per-acre context (40,000 acres in the United States), can be considered as a high-value crop (Cranberry Institute data). As a genetic resource for improving cultivated cranberry, wild *Vaccinium macrocarpon* populations can be found in peatlands, swamps and wet shores throughout eastern United States and Canada (Vander Kloet, 1988; Rodriguez-Bonilla et al., 2020). The niche of this species is similar to its wild relative *Vaccinium microcarpum* (2n = 2x = 24; also known as the small, swamp, or bog cranberry), although the latter is present throughout North America, but it is restricted to peatland environments (Vander Kloet, 1988). During the last decade, several studies have analyzed the genetic structure, diversity and geographic distribution all three wild species, *V. macrocarpon*, *V. microcarpum*, and the tetraploid *V. oxycoccos*, mostly through mitochondrial and nuclear SSR marker data (Smith et al., 2015; Zalapa et al., 2015; Schlautman et al., 2017b; Diaz-Garcia et al., 2019). Although these species are wild and have not been domesticated, their close shared ancestry, inter-species crossability, superior nutrient characteristics, improved fruit quality, and tolerance to abiotic stressors, could make them useful for breeding commercial cranberries (Vorsa et al., 2008).

Until recently, the lack of molecular tools limited the acceleration of cranberry cultivar development and genetic studies (Vorsa and Zalapa, 2020). Molecular tools in cranberry include multiple single sequence repeat (SSR) and single nucleotide polymorphism (SNP) based genetic maps (Georgi et al., 2013; Schlautman et al., 2015, 2017a; Covarrubias-Pazaran et al., 2016), a catalog of QTLs associated with yield related traits (Schlautman et al., 2015), fruit rot resistance (Daverdin et al., 2017), and fruit quality traits (Diaz-Garcia et al., 2018a,b), gene expression variation during fruit maturation (Sun et al., 2015), and a first draft, low coverage and highly fragmented genome (Polashock et al., 2014).

High quality nuclear genomes for crop species are now a prerequisite for advancing genetics and genomics research aimed at developing improved varieties (Benevenuto et al., 2019). Here, we applied Pacbio Sequel sequencing technology to generate a chromosome-level genome assembly of cranberry (*V. macrocarpon*), cultivar Stevens, and the first draft genome of its wild relative *V. microcarpum*. The recently developed molecular tools in cranberry, in conjunction with high-quality chromosome-level genome assemblies, will leverage cranberry downstream genetic analysis and support cultivar development and deployment.

## Materials and Methods

### Plant Material

For cranberry, we used the commercial cultivar Stevens, which was derived from two wild selections (McFarlin and Potter’s Favorite) (Vorsa and Zalapa, 2020). For *V. microcarpum*, we used an accession provided by N. Vorsa (Rutgers University), originally collected in southern Alaska (Mahy et al., 2000). Distinctive phenotypic characteristics between these two species and the similarity in their cytoplasmic genomes were further discussed in our previous study (Diaz-Garcia et al., 2019).

### Genome Sequencing and Assembly

High-molecular high-quality DNA from both species was extracted at Amplicon Express (Pullman, WA, United States). Thirteen single-molecule real-time (SMRT, insert size 30 Kb) cells of PacBio Sequel II (Pacific Biosciences of California, Inc., CA, United States) for cranberry and two for *V. microcarpum* were sequenced at The DNA Technologies and Expression Analysis Cores of the University of California-Davis (Davis, CA, United States). Illumina sequencing was carried out at the Biotech Center of the University of Wisconsin-Madison (Madison, WI, United States), using two PE libraries with insert sizes of 192.1 and 327.5 bp. Illumina sequence data was processed to filter out low-quality reads, remove adapters, and merge. PacBio sequencing data was assembled with Canu’s (Koren et al., 2017) automatic pipeline and corrected using the Illumina data with Pilon (Walker et al., 2014). Because the expected high heterozygosity level in both cranberry and *V. microcarpum*, we performed scaffolding with Redundans (Pryszcz and Gabaldón, 2016).

### Linkage Mapping and Cranberry Genome Scaffolding

Scaffolding in cranberry was based on four parental linkage maps derived from two previously studied mapping populations, CNJ02 and GRYG (Covarrubias-Pazaran et al., 2016; Schlautman et al., 2017a). Both populations were genotyped using genotyping-by-sequencing at the Cornell University Biotechnology Resource Center. Raw reads derived from GBS were used to call SNP markers in TASSEL v5 (Bradbury et al., 2007) using our new genome assembly as reference. SNP variants with less than five or more than 1,000 reads were removed. Biallelic SNP markers were then categorized according with the R package onemap (Margarido et al., 2007) nomenclature; D1.10 for markers in which parent 1 is heterozygous and parent 2 is homozygous (i.e., ABxAA), D2.15 for the opposite (AAxAB), and B3.7 for markers in which both parents are heterozygous (ABxAB). Then, the SNP markers were processed with the R package BatchMap (Schiffthaler et al., 2017), which is a parallelizable version of onemap. In BatchMap, we first identified marker bins, and then proceeded with the marker grouping (LOD = 18 for CNJ02 and 30 for GRYG) until obtaining 12 linkage groups per population. Subsequently, we split each linkage group into parental linkage groups based on the marker type (D1.10 and D2.15 markers going to different parental linkage groups, whereas B3.7 were transformed into parental-like versions which were subsequently used as anchor points between parental maps). Markers showing segregation distortion (*P* < 0.01, *X*^2^ test) were removed. Independently for each parental linkage group, we performed marker ordering and genetic distance computation, which then was corrected using a combination of ABHgenotypeR and an autoencoder generated with simulated data in kerasR (Arnold, 2017). After genotype correction, we recalculated genetic distance using the Kosambi function. In the end, we produced four parental maps composed of 12 linkage groups each which were inputted in ALLMAPS (Tang et al., 2015) for pseudomolecule construction. Collinearity between parental linkage maps was examined visually and using Spearman correlation. Chimeric scaffolds were identified and split in ALLMAPS (markers of the same scaffold mapping to different linkage groups) prior to the execution of the scaffolding step. Then, SNP marker calling and parental linkage map construction was repeated based on the new chimeric-free genome assembly (similar as above), and proceeded with pseudomolecule construction. A detailed flowchart with all the steps for sequence data generation, assembly, contig polishing and scaffolding, is presented in Supplementary Figure S1.

### Genome Annotation

RepeatModeler v2.0.1 (Smit and Hubley, 2008) was used to *de novo* model two independent repeat libraries based on the cranberry and *V. microcarpum* assemblies. Repeat elements were identified by RepeatMasker v4.1.0 (Smit, 2004) using RMBlastn v2.10.0 and the combined database of Dfam v3.1 (Wheeler et al., 2013) and Repbase v20181026 (Jurka et al., 2005).

Cranberry and *V. microcarpum* gene model prediction was carried out using the MAKER pipeline v.2.32 (Holt and Yandell, 2011), which includes *ab initio*, homology-based and RNA-seq assisted gene prediction. For the homology-based prediction we used peptide sequences from *Solanum lycopersicoides* (ITAG3.2) (Tomato Genome Consortium, 2012), *Helianthus annuus* (HanXRQ v 1.2) (Badouin et al., 2017), *Amaranthus hypochondriacus* (v1.0) *(Clouse et al., 2016)* and *Arabidopsis thaliana* (Araport11) (Cheng et al., 2017). For the RNA-seq assisted gene prediction, we reassembled published cranberry RNA-seq data from BioProject ids PRJNA246586 (Polashock et al., 2014) and PRJNA260125 (Sun et al., 2015) using Trinity v2.8.5 (Grabherr et al., 2011) with jaccard_clip parameter. MAKER *ab initio* training was performed using Augustus v3.2.1 (Stanke et al., 2006) with BUSCO-trained parameters using initial HMM model of embryophyta odb9 dataset (Simão et al., 2015) and a gene set with the best gene models based on a) Annotation Edit Distance (AED) scores < 0.25 and b) genes have to be at a distance of 1,000 bp from each other. A first round of MAKER was computed to construct gene models directly from both aligned transcript sequences and reference proteins. Then, three additional rounds of annotation using MAKER with Augustus and evidence build (proteins and transcripts) was performed to create an *ab initio* evidence-driven gene build. The functional inference for genes and transcripts were performed using the translated CDS features of each coding transcript. Each predicted protein sequences were blasted against the Uniprot/Swissprot database (The UniProt Consortium, 2018) to retrieve the gene name and the protein function as well as against InterProscan v5.44.76.0 (Jones et al., 2014) to retrieve Gene Ontology and domain information. Only blast hits with an *E*-value < 10E-6 were taken into account for gene name inference.

Finally, microRNAs, snoRNAs, tRNAs, and rRNAs were searched in the cranberry genome assembly using INFERNAL 1.1.3 (Nawrocki and Eddy, 2013) against the Rfam database 14.1 (Kalvari et al., 2018). Gene, repeat and ncRNA densities were visualized in Circos (Krzywinski et al., 2009).

### Phylome Construction

Protein sequences from 25 phylogenetic closely related species to *V. macrocarpon* were selected and downloaded for a phylogenetic analysis (Supplementary Table S7). The resulting sets of protein sequences were complemented with the protein sequences of *V. macrocarpon* and *V. microcarpum*, resulting in a final dataset of 1,385,343 protein sequences, distributed among the 27 species. The inference of the species tree was performed using the STAG method as it was implemented in the OrthoFinder program (Emms and Kelly, 2019).

Divergence times were estimated by first identifying orthologs from *Solanum lycopersicum*, *Actinidia chinensis*, *A*. *eriantha*, *Rhododendron delavayi*, *V*. *corymbosum*, cranberry and *V*. *microcarpum* with OrthoFinder. The divergence times were inferred by using RelTime as implemented in MEGA X (Tamura et al., 2012; Kumar et al., 2018). The following calibrations, obtained from TimeTree^1^ were used: *A*. *eriantha* versus *V*. *macrocarpon* minimal divergence time 52 Mya and maximal divergence time 96 Mya; *R*. *delavayi* versus *V*. *macrocarpon* minimal divergence time 30 Mya and maximal divergence time 89 Mya; finally, the divergence time from *A*. *chinensis* versus *A*. *eriantha* (minimal divergence time 3.2 Mya and maximal divergence time 3.4 Mya) was taken from Tang et al. (2019).

### Genome Synteny

Similar to previous studies (Iorizzo et al., 2016), we used a classical synonymous substitutions per site (Ks) age distribution analysis to study the evolution of the cranberry genome. All-to-all BLASTP analyses of proteins were performed between cranberry, tetraploid blueberry, *V. microcarpum*, carrot, tomato, kiwifruit, and grape, as well as within each of these species. BLASTP hits were filtered using 10E-3. Then, all-to-all BLASTP results were used to identify syntenic regions within and between species in MCScanX (Wang et al., 2012) with the following parameters: minimum number of genes per syntenic block = 5, *E*-value = 10E-5, gap penalty = −1, maximum number of gaps = 25, final score = 50. Collinearity within and between genomes were visualized using dot plots. All duplicated genes within the syntenic blocks were extracted and used to calculate the Ks values with MCScanX downstream tool add_ka_and_ks_to_collinearity.pl.

### Identification of Candidate Genes Related to Anthocyanin Biosynthesis

Cranberry structural genes involved in the anthocyanin biosynthesis pathway were manually curated. Additionally, anthocyanin biosynthesis-related transcription factors from the bHLH, MYB-HB-like, MYB-SANT and WD40-like families were predicted using the online platform plantTFcat (Dai et al., 2013).

In a previous study, we quantified total anthocyanin content in the CNJ02 population (used here for scaffolding) and identified multiple QTLs in chromosome 3 (Diaz-Garcia et al., 2018b). Phenotypes from this study were strongly supported by multi-year multi-location data produced by both a standard wet lab-based total anthocyanin determination and a computer vision method. Identifying candidate genes associated with QTLs in chromosome 3 was difficult given the fragmented genome assembly available at the time (Polashock et al., 2014). For the present study, we reused the phenotypic data (BLUPs, best linear unbiased predictors) in an attempt to identify candidate genes related with anthocyanin accumulation in cranberry fruit. The CNJ02 SNP markers generated based on the new cranberry assembly and that where used for linkage map construction and scaffolding, were also used here for QTL mapping. QTL mapping was carried out using a pseudo-testcross approach in r/qtl (Broman et al., 2003). Marker-trait associations were called based on a permutation test with 1,000 replicates using the n.perm = 1,000 argument in the scanone function. Finally, 1.5-LOD support intervals were calculated and candidate genes within these regions were identified for further inspection.

We performed a phylogenetic analysis with the cranberry candidate genes and flavonoid-related R2R3 MYB sequences from different subgroups (SG4-SG6). The deduced amino acid sequences were aligned using MUSCLE (Edgar, 2004) within Geneious Prime. A maximum likelihood phylogenetic tree was generated with 1,000 bootstrap replicates in MEGA X (Kumar et al., 2018). The GenBank accessions included in the analysis are presented in Supplementary Table S1.

## Results

### *De novo* Genome Assemblies of Cultivated Cranberry and Its Wild Relative

**FIGURE 1:**
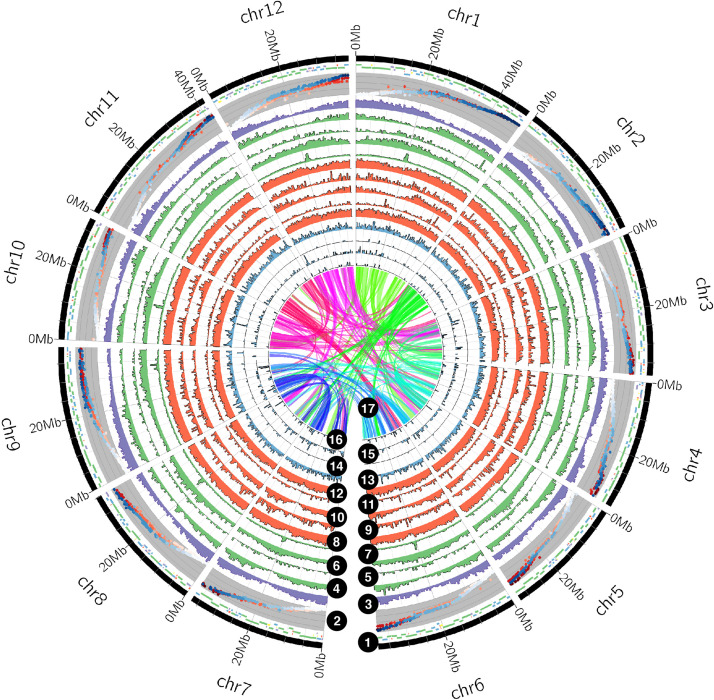
The American cranberry (*Vaccinium macrocarpon* Ait.) genome multidimensional landscape. Rings, from outside to inside, correspond to: (1) the tile plot depicts the anchored scaffolds into the final cranberry genome assembly, colored by the number of markers used for ordering and orienting (green > 10, blue > 5, purple > 2, yellow = 2, and red = 1); for visualization purposes, tiles are displayed among three concentric layers, however, there is no overlapping between them; (2) four overlapped scatter plots displaying genetic distances for the parental linkage maps used for scaffolding (CNJ02 and GRYG populations colored in red and blue, respectively); (3) gene density in 500 Kb non-overlapping bins; repeat density (500 Kb non-overlapping bins) by type, as follows: (4) SINEs, (5) ERVs, (6) LTR elements, (7) LINEs, (8) simple repeats, (9) satellites, (10) small RNAs (predicted by RepeatMasker), (11) rolling circles, and (12) DNA elements; non-coding RNA density (in 500 Kb non-overlapping bins) by type, as follows: (13) microRNAs, (14) tRNAs, (15) rRNAs, and (16) snoRNAs (predicted by Infernal). Links within and between chromosomes represent cranberry collinear blocks (17).

Using PacBio Sequel sequencing technology, we generated a chromosome-level genome assembly of the Stevens cranberry, the leading cultivar with 40% of acres planted worldwide (Vorsa and Zalapa, 2020). Our initial assembly (using 13 SMRT cells, for a 75.3 Gb yield and read length average of 10.57 Kb) consisted in 3,217 contigs (N50 = 2.18 Mb) spanning 642.17 Mb. Haplotypes were further collapsed using Redundans (Pryszcz and Gabaldón, 2016), which produced 812 scaffolds for a total length of 490.68 Mb (N50 = 1.36 Mb, GC content 37.88%). An analysis of k-mer frequencies with GenomeScope 2.0 (Ranallo-Benavidez et al., 2020) estimated a haploid genome size of 492.78 Mb (0.863–0.934% heterozygosity). Moreover, we generated chromosome-level pseudomolecules in ALLMAPS (Tang et al., 2015) using four newly developed parental linkage maps (4,875 unique marker positions) derived from two F_1_ cranberry mapping populations (CNJ02, *n* = 170; GRYG, *n* = 354) previously described (Covarrubias-Pazaran et al., 2016; Schlautman et al., 2017a). Marker collinearity between linkage groups among parental maps was nearly exact (mean Spearman’s *r* among chromosomes pairs = 0.97, standard deviation = 0.03; Supplementary Table S2 and Supplementary Figure S2). In total, 472 scaffolds (58%) were successfully anchored in 12 pseudomolecules, which represented 455 Mb (92.7% of the total genome assembly, Figure 1). From those, 332 scaffolds (88.7%) were oriented using at least two SNP markers per scaffold (12 scaffolds oriented based on 2-3 SNPs, 309 oriented based on at least four SNPs). Pseudomolecules produced by ALLMAPS (Tang et al., 2015) had a length between 33 (chromosome 10) and 48 Mb (chromosome 1, Supplementary Table S3). Chromosome naming was according to previous studies (Schlautman and Covarrubias-Pazaran, 2015; Schlautman et al., 2017a).

Using a similar strategy, we also generated a draft genome assembly of *V. microcarpum*, the closest wild relative of cranberry. For this species, we sequenced only 2 SMRT cells (14.27 Gb yield and read length average of 13.28 Kb), which resulted in 7,486 contigs (N50 = 149.76 Kb) with a total length of 764.21 Mb. Using Redundans, the assembly was further improved to 4,802 scaffolds (N50 = 176.33 Kb, GC content 38.14%) and total length of 622 Mb. Additional statistics are presented in Supplementary Table S4.

To assess genome assembly completeness, we compared cranberry and *V. microcarpum* assemblies to 1,614 Benchmarking Universal Single-Copy Orthologs (BUSCOs) (Simão et al., 2015). This analysis recovered 93.4 and 87.4% complete BUSCOs in the cranberry and *V. microcarpum* genomes, respectively (Supplementary Table S5). Moreover, based on the high long terminal repeat (LTR) Assembly Index (LAI) (Ou et al., 2018) scores of 17.57 and 13.60, the cranberry and *V. microcarpum* genome assemblies attained reference level qualities, respectively. Finally, 94.2% of previously published cranberry assembled transcripts (Sun et al., 2015) mapped to the cranberry genome confirmed its high quality.

### Gene Prediction and Annotation

We identified approximately 255 and 313 Mb (∼50% of each assembly) of repetitive sequences in the cranberry and *V. microcarpum* genomes, respectively (Supplementary Table S6). The content of repetitive sequences in both genomes reported here appears to be higher than the previous cranberry draft genome (39.5%) (Polashock et al., 2014); however, they are consistent with reports in other Ericaceae species (Colle et al., 2019; Soza et al., 2019; Wu et al., 2019). We found a large portion of the unclassified sequences in cranberry and *V. microcarpum* to be repetitive sequences (23.97 and 22.74%, respectively), which might be *Vaccinium* specific. Retrotransposons were the largest repeat category in the cranberry genome (∼17.8% of the assembly), among which the long terminal repeat (LTR) family was the most abundant (∼14.0% of the assembly). Within the LTR family, Copia and Gypsy represented the two most abundant subfamilies (∼5.0 and 8.3%, respectively). In addition, DNA transposons accounted for 6.6% of the genome assembly. For *V. microcarpum*, the classification of repetitive elements was similar to *V. macrocarpon*, 18.87% retroelements and 6.86% DNA transposons.

Using the cranberry RNA-seq data published in two previous studies (Grabherr et al., 2011; Polashock et al., 2014), integrated with *ab initio* gene predictions and homologous sequence searching, we predicted 23,532 protein-encoding genes in the cranberry assembly, with an average coding sequence length of 1,557 bp and 6.7 exons per gene. For *V. microcarpum*, we predicted 30,147 protein-encoding genes, with an average coding sequence length of 1,448 bp and 6.9 exons per gene. Among these genes, 83.37% (cranberry) and 81.87% (*V. microcarpum*) had significant similarities to sequences in the Uniprot/Swissprot database. Additionally, we annotated 50.19 (cranberry) and 47.70% (*V. microcarpum*) of the genes using the GO database. Furthermore, conserved domains in 78.26 (cranberry) and 74.70% (*V. microcarpum*) of the predicted protein sequences were identified by comparing them against the InterPro and Pfam databases. Finally, we identified 31 rRNA fragments, 478 tRNAs, 2,902 small nucleolar RNAs (snoRNAs), and 185 microRNAs (miRNAs) in the cranberry genome assembly (Figure 1 and Supplementary Table S7).

### Cranberry Genome Evolution

**FIGURE 2:**
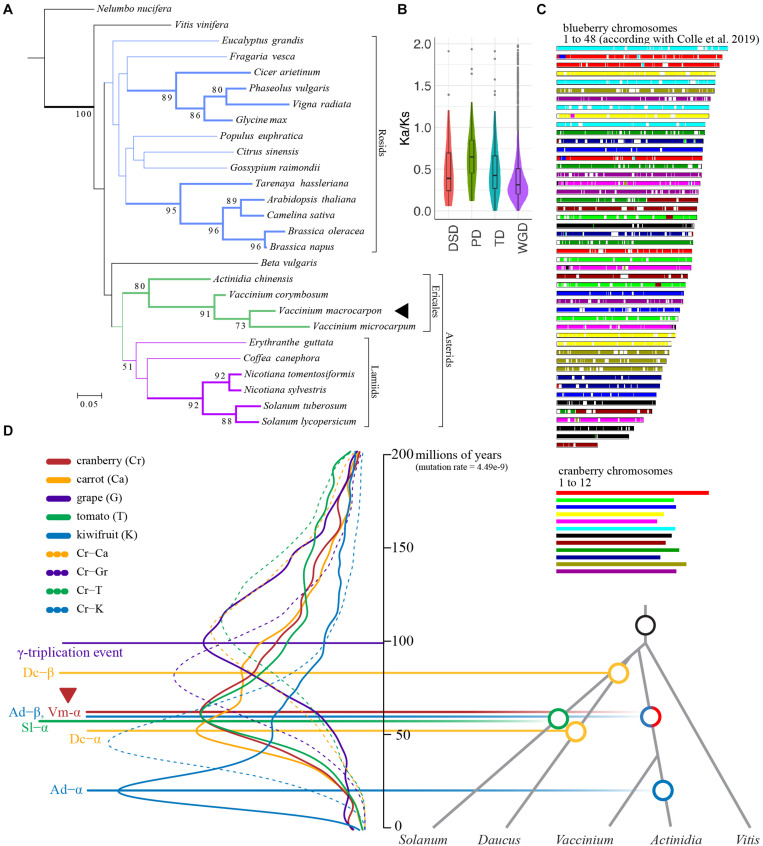
The American cranberry (*Vaccinium macrocarpon* Ait.) genome evolution. **(A)** Phylogenomic analysis for 27 species and 6,327 orthogroups. Only bootstrap values larger than 50 are shown. Nodes supported by bootstraps > 70 are shown as tick bars. **(B)** Ka/Ks ratio distributions of gene pairs grouped by four different types of duplication (WGD, whole genome duplication; TD, tandem duplication; PD, proximal duplication; DSD, dispersed duplication). In the boxplots, points represent outliers; the center line is the mean; lower and upper hinges are 25th and 75th percentiles. **(C)** Schematic representation of the collinearity between cranberry and tetraploid blueberry (chromosome sizes between species are not scaled). **(D)** Distribution of the synonymous substitution (Ks) rate for collinear genes from cranberry (*Vaccinium macrocarpon*), carrot (*Daucus carota*), grape (*Vitis vinifera*), tomato (*Solanum lycopersicum*) and kiwifruit (*Actinidia chinensis*). Solid and dashed lines represent intra and inter species comparisons, respectively; Ks estimates were converted to millions of years using *T* = *Ks/2r*, where *r* = 4.49^– 9^ (Wei et al., 2018; Wu et al., 2019).

A phylogenomic tree was inferred for 27 species and 6,327 orthogroups (Supplementary Tables S8, S9). In general, phylogenetic relationships between species exhibited in the tree are in agreement with previous studies (Vanneste et al., 2014; Figure 2A). The phylogeny recovered the monophyly of rosids and asterdis as well as that of ericales and lamiids within the asterids. However, well supported nodes (showing bootstrap > 70) were restricted to terminal taxa (these are shown as thick lines in Figure 2A). These include species from the genus *Nicotiana*, *Solanum* within the Lamiids, all the Ericales and two clades within the Rosids. To estimate divergence times, we identified 86 orthogroups for a smaller set of species, used RelTime (Tamura et al., 2012) as well as two calibrations derived from TimeTree (see Footnote 1). Molecular dating revealed that cranberry and *V. microcarpum* divergence occurred 4.5 Mya (3.0–6.7), following the divergence of their common ancestor and blueberry 10.4 Mya (8.1–13.4). Furthermore, molecular dating-based analysis suggested *Vaccinium* and *Actinidia* divergence occurred 52.1 Mya (52.0–63.3).

By comparing the cranberry genome with itself and other Eudict species, we identified a large number of syntenic blocks (Supplementary Table S10). Specifically, within cranberry we identified 5,136 collinear genes (22%) among 266 syntenic blocks (11.91 gene per block). The rest of the genes were classified as singletons (12%), dispersed (47%), proximal (5%), and tandem (15%). Higher synonymous-to-non-synonymous substitution rate ratios (Ka/Ks) were found for proximal duplicate gene pairs, suggesting an ongoing and continuous process for proximal duplications, stronger positive selection, and faster sequence divergence than genes produced by other duplication phenomena (Figure 2B), similar to what has been observed in other Ericales species (Yang et al., 2020). Comparison of cranberry against its wild relative (*V. microcarpum*), kiwifruit, tetraploid blueberry, tomato, grape and carrot yielded 20,163 (38.03%), 31,127 (49.15%), 64,919 (45.94%), 20,815 (39.36%), 17,165 (16.84%), and 22,728 (42.33%) collinear genes, respectively. Because of the close relationship between cranberry and blueberry, we identified an exceptional collinearity between chromosomes of both species (Figure 2B), an observation previously described (Schlautman et al., 2017c).

Synonymous substitutions per site (Ks) age distributions and synteny analysis unveiled evidence for a cranberry ancient polyploidization (the γ-triplication, a shared event among core eudicots), followed by a more recent whole genome duplication (WGD) event ∼61 millions of years ago (Figure 2C). This recent WGD, named here as Vm-α, is probably shared with the *A. chinensis* Ad-β event (Wu et al., 2019) that occurred in the Cretaceous-Paleogene (K-Pg) boundary (Van de Peer et al., 2017). According with our analysis, Ad-β WGD approximately co-occurred with the most recent *S. lycopersicum* WGT (Sl-α), also during the K-Pg boundary (Tomato Genome Consortium, 2012). Our analysis agreed with results from a comparative genomics study in *Rhododendron williamsianum* and related species in the Ericaceae family that found two shared WGDs (the γ-triplication and Ac-β events) among cranberry (the Vm-α event found here), *Camellia sinensis*, and *Rhododendron* (Soza et al., 2019). In relation to the evolutionary history of *Vaccinium* species, our molecular dating-based estimation for the divergence of cranberry-*V. microcarpum* ancestor and blueberry (10.4 Mya) agreed with a recent study (Wang et al., 2020) dating the most recent polyploidization event in tetraploid blueberry (approximately 9 Mya) little after divergence.

### Anthocyanin Biosynthesis and Identification of Candidate Genes

Anthocyanin content determines color in cranberry fruit, and due to its importance for human health, it is the main parameter of quality considered by the cranberry industry (Vorsa and Zalapa, 2020). The different types of anthocyanins in cranberry fruit are well known as well as their variation during fruit ripening (Wang et al., 2017); however, no candidate genes involved in anthocyanin biosynthesis and accumulation have been identified so far. Our gene model annotation pipeline allowed us to predict 18 unique structural genes involved in anthocyanin biosynthesis and accumulation in the cranberry genome (Supplementary Table S11). In terms of gene copy, 4-coumarate CoA ligase (13), *O*-methyltransferase and anthocyanidin 3-0-galactosyltransferase (8 each) genes were the most numerous. Based on our synteny analysis, dispersal (38%) and tandem (35%) duplication mechanisms were dominant among anthocyanin-related structural genes (Supplementary Table S12). Virtually all key structural genes involved in the anthocyanin biosynthesis pathway were identified (Jaakola et al., 2002; Sun et al., 2015).

Similarly, genome-wide prediction of transcription factors (TF) using plantTFcat (Dai et al., 2013) identified 627 genes associated with four well-known anthocyanin biosynthesis-related gene families; these are bHLH (113 TF identified), MYB-HB-like (222), MYB-SANT (25) and WD40-like (267, Supplementary Table S13). The distribution of anthocyanin-related TF among cranberry chromosomes was uniform (from 37 to 64). Only 40 TF (6%) were located among unanchored cranberry scaffolds. It has been showed that members of the MYB-HB-like family (i.e., R2R3MYB) are key regulators of the anthocyanin biosynthesis (Plunkett et al., 2018). As a result of our analysis with cranberry orthologous, cranberry TF belonging to the MYB-HB-like family (222) were grouped among 103 orthogroups. From those, 60 groups had at least a gene from each of the species included in the analysis (27). Nine orthogroups were *Vaccinium*-specific (contained genes only from at least two out of the three *Vaccinium* species included in the analysis). Cranberry bHLH TF (113), which are also known to be involved in anthocyanin regulation, were grouped among 67 orthologous groups, from which 33 contained genes from each of the species in the analysis; six orthogroups were *Vaccinium*-specific. Among the 627 TFs, 31% were retained after WGD events, whereas 52% were the result of dispersed duplication (Supplementary Table S12).

**FIGURE 3:**
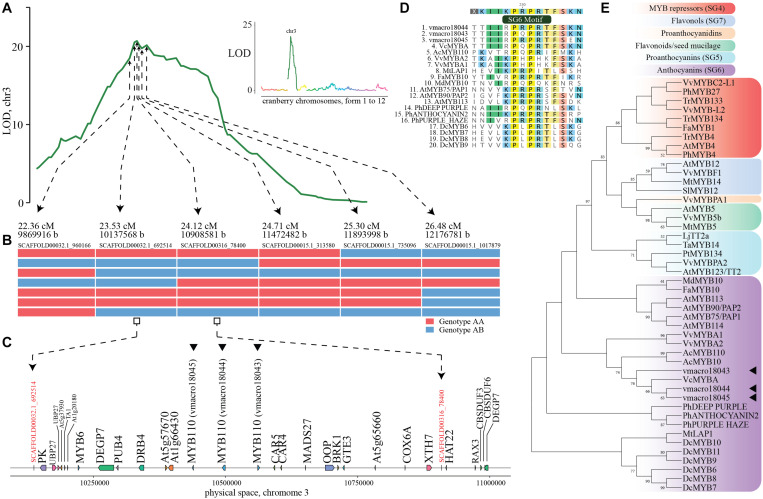
Identification of candidate genes for fruit anthocyanin biosynthesis in cranberry (*Vaccinium macrocarpon* Ait.) **(A)** LOD score profile for total anthocyanin content in the CNJ02 population; in the small panel, LOD profile is shown for the entire genome, whereas the main panel emphasizes chromosome 3. **(B)** Recombinant haplotypes delimiting the genome region of maximum LOD score (within the 1.5-LOD interval). **(C)** Schematic representation of gene content within two contiguous markers at maximum LOD score; anthocyanin related genes (vmacro18043-vmacro18045) are marked with a triangle. **(D)** Multiple alignment of cranberry candidate genes and R2R3MYB anthocyanin-related transcription factors in other species that present the subgroup 6 (SG6) motif. **(E)** Maximum likelihood phylogenetic tree of cranberry candidate genes and characterized flavonoid-related R2R3MYB sequences from other species. Node support > 50% from 1,000 bootstrap replicates is shown.

Complementarily, we employed QTL mapping using multi-year phenotypic data from our previous study (using the CNJ02 population, *n* = 170, Supplementary Table S14) in which multiple QTL in chromosome 3 were associated with anthocyanin variation (Diaz-Garcia et al., 2018b). Anthocyanin content in fruit showed great variation among genotypes, from 16.05 mg/100 FW (fruit weight), to 28.88 mg/100 FW. Genomic heritability for this trait was 0.36. Our QTL mapping analysis performed here confirmed a large QTL in position 24.12 cM of chromosome 3 (LOD = 20.69, explained variance = 51.16%), with physical coordinate 10908581 (Figure 3A). Within the 1.5-LOD interval (22.36–28.24 cM, that corresponds to coordinates 9869916-12333384) and a conserved recombination block among 169/170 individuals (Figure 3B), we identified a cluster of three genes similar to *A. chinensis* R2R3 MYB110 (genes vmacro18045:10437125-10439365, vmacro18044:10493992-10499550 and vmacro18043:10561041-10564609, Figure 3C).

We performed a phylogenetic analysis on vmacro18043-vmacro18045 genes against well-characterized flavonoid-related R2R3 MYB sequences from other species. Sequences included proanthocyanidin (subgroup 5, or SG5), anthocyanin (SG6) and flavonols (SG7) activators, as well as R2R3 MYB repressors (SG4). A multiple alignment of the deduced amino acid sequences of the R2R3 MYB sequences revealed that two cranberry candidate genes (vmacro18044 and vmacro18045) presented 5/6 residues of the subgroup-6 motif in the variable C-terminal region, which is not uncommon among recognized SG6 R2R3 MYB anthocyanin regulators (Figure 3D; Plunkett et al., 2018). In the phylogenetic tree, all three cranberry candidate genes were grouped within members of the SG6 anthocyanin biosynthesis activators. *Vaccinium corymbosum* MYBA, as well as both *A. chinensis* MYB10 and MYB110, which have been directly associated with anthocyanin biosynthesis regulation, were included closely within the same clade (Figure 3E). Orthologous groups computed as part of our phylome analysis grouped vmacro18044 and vmacro18045 into OG0000979, which contains 153 genes among 24 species. The vmacro18043 gene was included in OG0013302 orthologous group, which contains 30 genes among six species, mostly from blueberry (21). Furthermore, gene expression data in cranberry show that both vmacro18044 and vmacro18045 are expressed in fruit (Sun et al., 2015). All three candidate genes were not detected as part of those retained after polyploidization events, but as a result of tandem duplications, which is a common duplication mechanism for these gene families (Matus et al., 2008; Du et al., 2012; Supplementary Table S12).

## Discussion

The Ericales order comprises more than 8,000 species among them cranberry, blueberry and kiwifruit stand out as the most important fruit crops. Genome sequences have been generated for all three species before (Huang et al., 2013; Polashock et al., 2014; Gupta et al., 2015; Colle et al., 2019; Wu et al., 2019); however, only blueberry and kiwifruit have chromosome-level assemblies. High-quality chromosome-scale reference genomes benefit downstream genetic analysis and accelerate the genetic improvement of crops (Benevenuto et al., 2019). For example, high-quality genomes improve the precision regarding QTL location and action, increase the chances of identifying candidate genes (a larger gene space to compare with), and help the dissection of the genetic architecture governing phenotypic variation (Benevenuto et al., 2019). Marker assisted breeding approaches, especially in fruit crops (larger selection cycles), are considerably benefited when highly reliable marker probes (i.e., phenotype of interest cosegregates perfectly with genetic marker) are incorporated into their selection/prediction pipelines. Furthermore, high-quality genomes are of great importance when reducing genotyping costs in association mapping International Wheat Genome Sequencing Consortium [IWGSC], Appels et al. (2018) and genomic prediction (de Los Campos et al., 2013) studies by making more efficient the use of molecular probes.

In this study, we present the first chromosome-scale cranberry genome assembly based on long-read Pacbio Sequel II technology. Out of the 492 Mb predicted genome size, 455 Mb (out the 490 Mb assembly) were anchored in 12 chromosomes, which is comparable with other recently published genomes. In terms of assembled sequences (812 scaffolds, 490 Mb), our cranberry assembly represents an increase of 16% compared with the previous draft genome and a decrease of 99.96% in the number of pieces (Polashock et al., 2014). Regarding gene annotation, we predicted 23,532 gene models, a low number compared with other eudicot genomes (carrot v2.0 32,113; grape Genoscope.12 × 26,346; kiwifruit v3.0 40,464; *Camellia sinensis* v1.0 33,932; tomato iTAGv2.3 34,727). In the previous cranberry draft genome, approximately 30,000 genes were predicted; however, only 13,170 (36%) were supported by transcriptome data. Genome assemblies of *R. williamsianum* (532 Mb genome assembly) (Soza et al., 2019) and *R. delavay* (695 Mb genome assembly) (Zhang et al., 2017), two species within the Ericaceae family, have reported 23,559 and 32,938 predicted genes, respectively.

Here, we dated a whole genome duplication event in the Ericales lineage 61 Mya. This WGD event is shared among members of the Ericaceae and Actinidiaceae families as also suggested previously (Soza et al., 2019; Wu et al., 2019; Yang et al., 2020). Many studies have documented the co-occurrence of WGDs during the K-Pg boundary, a period in which most plant lineages started to diversify considerably (Van de Peer et al., 2017). Most of the evidence so far suggests that all Ericales experienced a common WGD during the K-Pg period, and from that, selected species have experienced subsequent additional WGDs. Compared with tetraploid blueberry, *Camelia*, and *Actinidia*, cranberry, *V. microcarpum*, as well as other species of the *Rhododendron* genera, have not suffered more recent whole genome duplications.

Recently, cranberry breeders have emphasized the creation of better-tasting, low-acid, and high-sugar content cranberry varieties (Fong et al., 2020; Vorsa and Zalapa, 2020). However, to date, most of the cranberry cultivars available are being used for processing (e.g., juice, sweetened and dried cranberries, Etc.), mainly because fresh fruit still lacks attractive attributes in terms of flavor for its consumption as fresh. Several *Vaccinium* species are characterized by a remarkable genome collinearity (Schlautman et al., 2017c), which allows cross-compatibility in interspecific hybridization and facilitates the introgression of desirable traits into cranberry. Particularly, *V. microcarpum* has a circumboreal distribution and grows in higher latitudes than cranberry, therefore, exhibits an enhanced resistance to low temperatures. Additionally, this wild relative could be used as a bridge to reach more distant *Vaccinium* species such as blueberries, which possess desirable traits such as lower acidity and increase sugar content (Vorsa et al., 2008). Having a comprehensive understanding of the genome architecture, gene catalog, and metabolite regulation of cranberry and their close relatives will definitely accelerate its genetic improvement.

One of the most attractive attributes of cranberry fruit is its high anthocyanin content. So far, multiple QTLs have been associated with variation on this trait (Diaz-Garcia et al., 2018b); however, although anthocyanin biosynthesis actors have been well characterized in closely related species such as blueberry (Plunkett et al., 2018) and kiwifruit (Li et al., 2017), no candidate genes have been reported for cranberry. Here, we described a list of 18 multi-copy structural genes involved in the anthocyanin biosynthetic pathway, as well as more than 600 transcription factors belonging to four known families related with anthocyanin biosynthesis regulation. Most of the cranberry TF in these four families have orthologous genes among multiple species, some of which have been recognized as key anthocyanin regulators. Using previously published QTL mapping data (Diaz-Garcia et al., 2018b), we identified a cluster of three R2R3 MYB transcription factors belonging to the subgroup 6, which includes well-characterized anthocyanin biosynthesis regulators in multiple species. Two of the candidate genes, vmacro18044 and vmacro18045, exhibit extremely high similarity with blueberry MYBA TF, which is known to directly activate anthocyanin biosynthesis (Plunkett et al., 2018). Moreover, transcriptome evidence showed that these two genes are expressed in cranberry fruit (Sun et al., 2015). Anthocyanin accumulation variation in the mapping population was limited, and all the individuals did produce anthocyanins. Therefore, we believe vmacro18044 and vmacro18045 act as anthocyanin modulators and not as on/off switches. To the best of our knowledge, this is the first time candidate genes for anthocyanin regulation are reported for cranberry.

The chromosome-level cranberry genome, and the draft genome of its close wild relative *V. microcarpum*, provide a much-needed resource for further investigation of the genetic architecture underlying phenotypic variation. These genomes will support cranberry improvement efforts by facilitating the discovery of novel marker-trait associations useful in marker-based breeding strategies, the fine-mapping of candidate genes, and the creation of better genomic selection frameworks.

## Data Availability Statement

Genome assemblies and annotation data have been deposited in the Genome Database for Vaccinium (https://www.vaccinium.org), under accession numbers GDV20001 (*Vaccinium macrocarpon* Ait.) and GDV20002 (*Vaccinium microcarpum* L.).

## Author Contributions

LD-G and JZ conceived and supervised the project. JZ provided the plant materials for sequencing and genetic mapping. LD-G performed the sequencing and assembly. LG-O performed the annotation. LD-G, LG-O, MG-R, LD, and MI performed the evolution analysis. LD-G and MI performed anthocyanin analysis. LD and JZ organized the manuscript. LD-G, LG-O, LD, MI, and JZ wrote the manuscript. All authors read, edited, and approved the final manuscript.

## Conflict of Interest

The authors declare that the research was conducted in the absence of any commercial or financial relationships that could be construed as a potential conflict of interest.

## Footnotes

1 http://www.timetree.org

## Supplementary Material

Supplementary Figure 1 Plant material, sequencing resources, and methods for sequence data generation, assembly, polishing, and scaffolding of the cranberry and *Vaccinium microcarpum* genome assemblies.

Supplementary Figure 2 Parental linkage maps for the CNJ02 and GRYG populations.
