# Supplementary figures and images for: Chromosome-Level Genome Assembly of the American Cranberry (Vaccinium macrocarpon Ait.) and Its Wild Relative Vaccinium microcarpum
Source: Front Plant Sci. 2021 Feb 10;12:633310. doi: 10.3389/fpls.2021.633310 (PMC7902871; doi:10.3389/fpls.2021.633310)

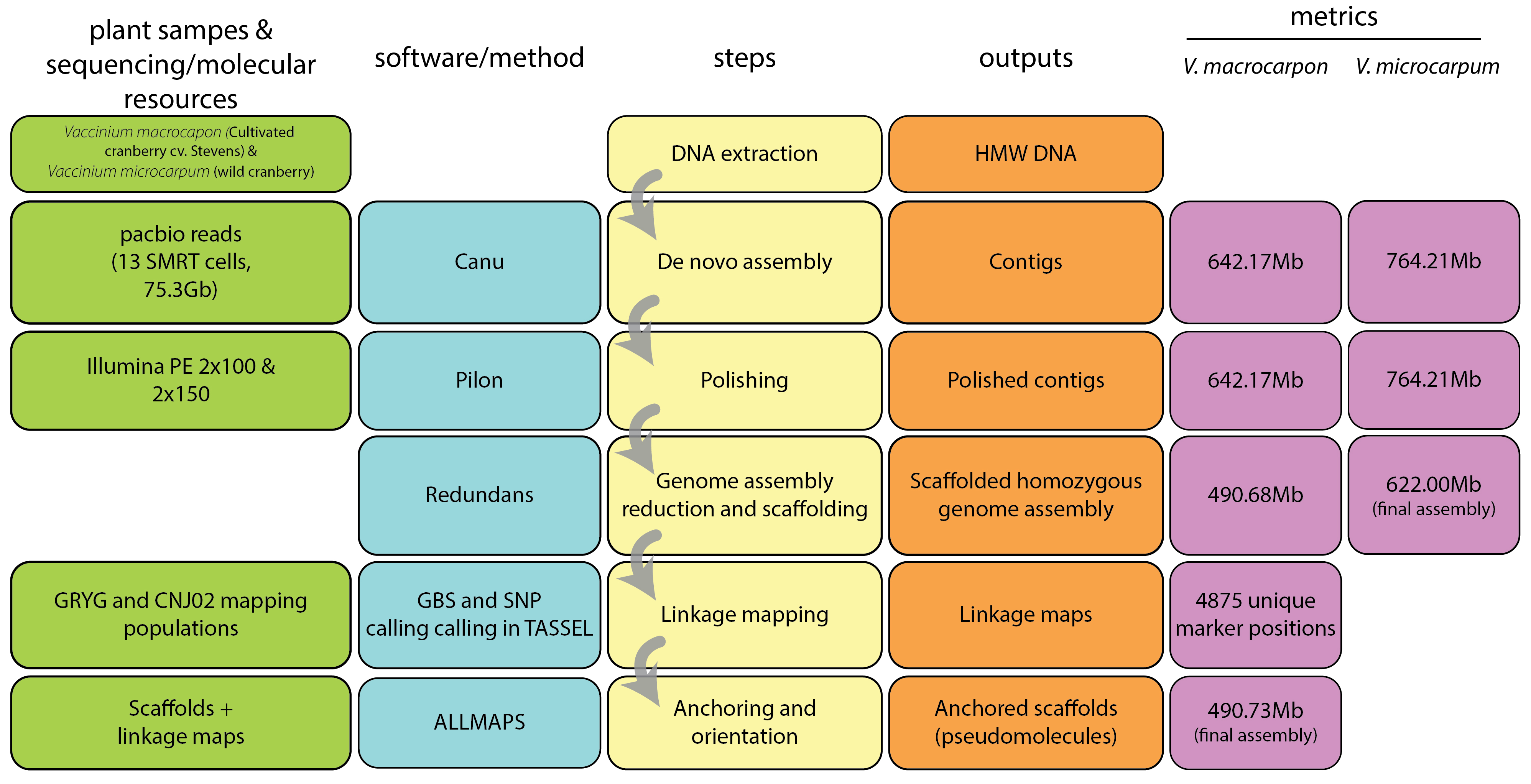

Supplement: Supplementary Figure 1 — Plant material, sequencing resources, and methods for sequence data generation, assembly, polishing, and scaffolding of the cranberry and Vaccinium microcarpum genome assemblies. [file Image_1.JPEG]

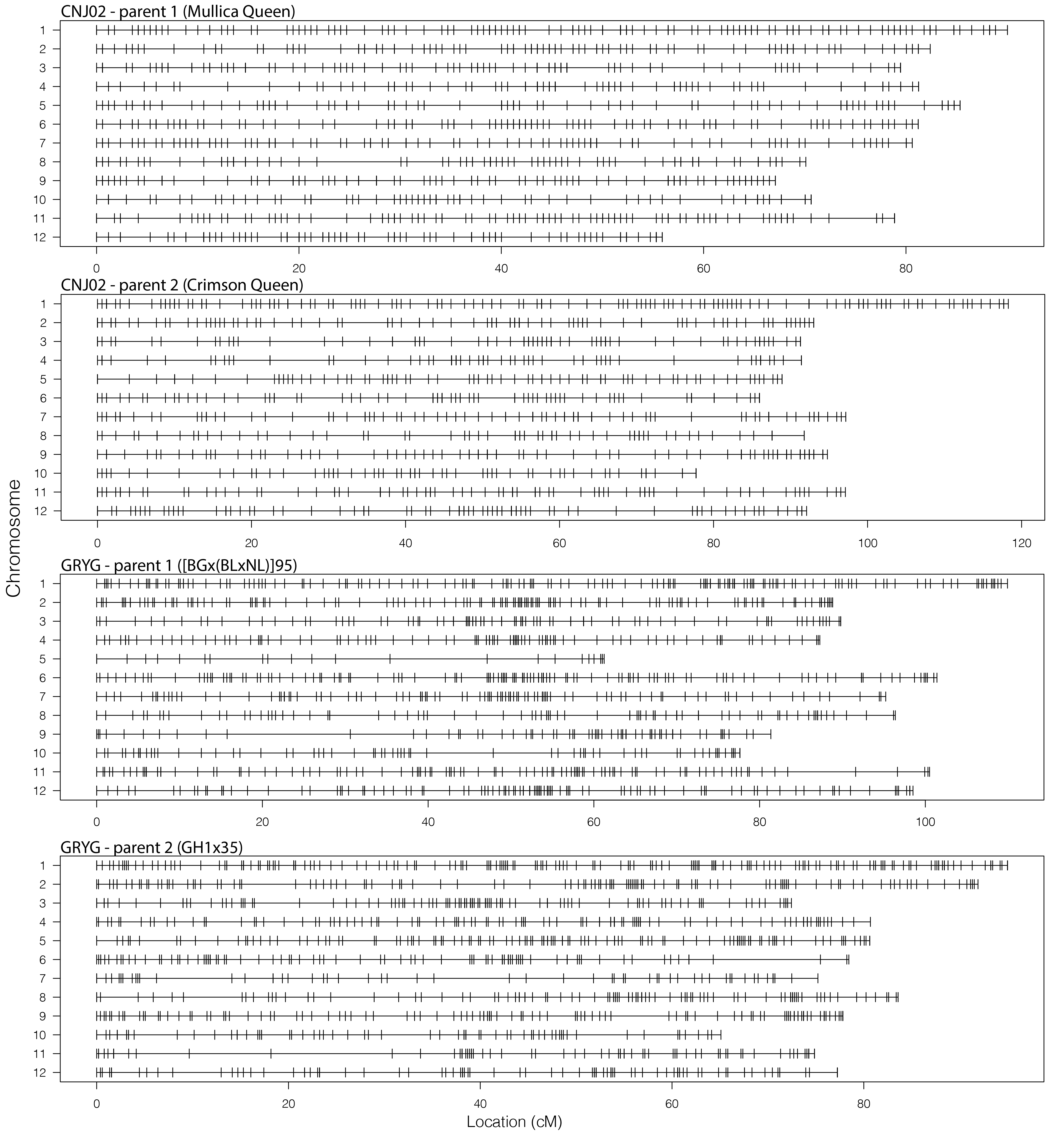

Supplement: Supplementary Figure 2 — Parental linkage maps for the CNJ02 and GRYG populations. [file Image_2.JPEG]
